# The role of implementation science training in global health: from the perspective of graduates of the field’s first dedicated doctoral program

**DOI:** 10.3402/gha.v9.31899

**Published:** 2016-11-14

**Authors:** Arianna R. Means, David E. Phillips, Grégoire Lurton, Anne Njoroge, Sabine M. Furere, Rong Liu, Wisal M. Hassan, Xiaochen Dai, Orvalho Augusto, Peter Cherutich, Gloria Ikilezi, Caroline Soi, Dong (Roman) Xu, Christopher G. Kemp

**Affiliations:** Department of Global Health, University of Washington, Seattle, WA, USA

**Keywords:** global health, implementation science, education, training, curriculum

## Abstract

Bridging the ‘know-do gap’ is an enormous challenge for global health practitioners. They must be able to understand local health dynamics within the operational and social contexts that engender them, test and adjust approaches to implementation in collaboration with communities and stakeholders, interpret data to inform policy decisions, and design adaptive and resilient health systems at scale. These skills and methods have been formalized within the nascent field of Implementation Science (IS). As graduates of the world's first PhD program dedicated explicitly to IS, we have a unique perspective on the value of IS and the training, knowledge, and skills essential to bridging the ‘know-do gap’. In this article, we describe the philosophy and curricula at the core of our program, outline the methods vital to IS in a global health context, and detail the role that we believe IS will increasingly play in global health practice. At this junction of enormous challenges and opportunities, we believe that IS offers the necessary tools for global health professionals to address complex problems in context and raises the bar of success for the global health programs of the future.

## Introduction

Global health is at a crossroads. The technical, cultural, and political changes of the previous two centuries have led to new economic, social, and health inequities within and across countries. These same changes have given rise to the resources, innovations, and interventions to address these inequities and practitioners who are dedicated to doing so. Global health practitioners are adept at developing solutions for public health problems through carefully controlled research, although our weakness has been in taking the crucial next step: translating those solutions into scalable and equitable health improvements while disseminating knowledge and experiences within our community.

Global health practitioners require interdisciplinary skills and methods to take this step. They must be able to understand local health dynamics within the operational and social contexts that engender them, test and adjust approaches to implementation in collaboration with communities and stakeholders, interpret data to inform policy decisions, and design adaptive health systems at scale. These skills and methods have been collected and formalized within the nascent field of Implementation Science (IS). IS builds upon, but is distinct from, complementary disciplines such as health services research. IS is a systematic, scientific approach to identifying and delivering quality health care and effective health programs to people who need it, with speed, fidelity, efficiency, and coverage. As graduates of the world's first PhD program dedicated to IS, the authors of this article have a unique perspective on the role of IS. We aim to share our insights regarding the training, knowledge, and skills that are essential for applied IS research and practice.

## The value of IS in modern global health practice

Global health programs may fail to meet their stated goals for several reasons. One reason is intervention failure, when interventions are ineffective. Another is implementation failure, when effective interventions are incorrectly implemented (1). The latter may occur when an intervention is inappropriate or inappropriately tailored to a setting. While the intervention has typically been the scientific emphasis in global health research, both are opportunities for IS.

For example, in 2012, the World Health Organization (WHO) endorsed the Option B+ policy whereby HIV-positive pregnant women are encouraged to initiate antiretroviral treatment during pregnancy, regardless of CD4+ cell counts, and maintain treatment for life (2). The country-specific challenges to implementing such a policy, such as linking antenatal and HIV care, were numerous. Public health practitioners had to decide how to implement or adapt an efficacious intervention across deeply heterogeneous settings (3). To reduce implementation failure, practitioners have applied a variety of IS methods. In Malawi, IS practitioners tested health worker mentorship, facility-level quality improvement interventions, and couple's HIV testing to improve Option B+ uptake, subsequently influencing the national rollout of the program (3). In Mozambique, researchers used formative research to guide the design of a stepped wedge trial to study the effects of antenatal workflow modifications, adherence, and retention packages (4). In Zambia, researchers used a quasi-experimental design at government antenatal clinics to test methods for overcoming programmatic barriers including decentralized care, disease-stage assessment delays, and loss to follow-up (5). And in South Africa, researchers assessed the feasibility of a mobile phone-based case manager intervention to support treatment initiation (6). These IS methods facilitated effective Option B+, introduction and scale-up with continuous monitoring for and mitigation of potential implementation failure.

## Essential IS skills and methods

IS aims to bridge the ‘know-do gap’ by applying methodologically rigorous approaches to 1) generating and synthesizing population-level evidence within context, 2) testing interventions informed by contextual evidence, 3) translating appropriate findings into practice and, 4) continuing the evidence generation cycle. Interdisciplinary skills and methods are essential for these approaches, as described below (7, 8).

Poor data, missing data, or a complete lack of data is a common challenge. IS practitioners must be able to identify evidence that is scientifically and structurally relevant to a particular health problem, leverage routine data as much as possible, and generate evidence when it is absent. Data quality assessment, data synthesis, and modeling techniques allow practitioners to learn even from noisy and incomplete data. New information technologies and routine data collection systems allow IS practitioners to collect evidence, with ever shorter delays in data collection and analysis processes (9). In addition, advanced qualitative research methods help researchers build theories for how and why an intervention may be necessary and appropriate.

Once relevant data are analyzed, interventions that are effective, appropriate, or scalable should be carefully evaluated. Pragmatic, non-randomized, or quasi-experimental study designs – including stepped wedge cluster randomization, interrupted time series, and regression discontinuity – allow robust evaluation of interventions in real-world settings (10–13). These designs can evaluate health system interventions without requiring the use of expensive experimental designs that are internally valid but uninformative for program implementation. The so-called ‘hybrid’ study designs have utility in this regard: simultaneously testing intervention efficacy while directly informing approaches to implementation and dissemination (14). A defining characteristic of IS is thus its focus on both health and implementation outcomes. IS specialists must be able to design studies that can inform how an intervention should be carried out, and not only whether it is effective.

IS practitioners should also be able to support dissemination and translation of evidence into policy and practice. They may ensure that new evidence is relevant to local context, appropriate local stakeholders are involved, a health systems perspective is used to assess broader influence of an intervention, and robust monitoring and evaluation systems are available to assess the impact and iteratively test the performance. To this end, practitioners must be adept in the use of the theoretical frameworks that form the backbone of IS. The most prominent of these is the Consolidated Framework for Implementation Research (CFIR), which helps identify factors that predict implementation success in order to develop more effective health programs. The CFIR is a meta-theoretical framework, synthesizing the range of terminologies, definitions, and constructs related to implementation into a single framework of factors that influence implementation outcomes (15). By organizing their work around such frameworks, IS practitioners will promote the replicability and generalizability of their findings.

## The role of formal IS training

IS skillsets and training are relevant to a number of pressing issues in global public health and are increasingly recognized by health and development institutions. For example, the World Bank launched an initiative in 2013 to improve the ‘science of delivery’, while the WHO launched the ‘implementation research platform’ in 2010 (16). Given the increased demand for and complexity of advanced IS skills, an implementation scientist must be trained on a spectrum of skillsets. With today's unprecedented exchange of knowledge, these skillsets should come from a variety of disciplines and fields.

The Department of Global Health at the University of Washington (Seattle, WA, USA) launched a PhD program in Implementation Science and Health Metrics in 2012. As students in this innovative program, we are learning the technical and applied skillsets necessary for bridging the ‘know-do gap’. The core curriculum draws from and adapts skillsets from epidemiology, biostatistics, qualitative research, policy analysis, health services research, quantitative impact evaluation, economics, systems engineering, anthropology, computer science, and many more.

IS training has a broader scope than that of ‘standard’ public health training. While traditional public health practitioners place scientific emphasis on preventing intervention failure, IS specialists broaden their attention to study and prevent implementation failure. Experimenting with both interventions and implementation strategies demands a skillset that is unique for two reasons.

First, IS requires a multidisciplinary approach to generating high-quality evidence and integrating it into practice. This multidisciplinary approach draws from the scientific toolset most fit for the purpose of ensuring that a proposed intervention is unequivocally effective and socially acceptable. IS practitioners are not necessarily experts in all disciplines, but must be trained to work and synthesize information across disciplines. Accordingly, the authors’ research focuses range from applying novel study designs to test the rollout of partner notification services for HIV to pairing economic analyses with qualitative research to identify best practices in integrated neglected tropical disease programming.

Second, IS requires a broad health systems perspective to understand the multisectoral ancillary effects of health interventions or policies. IS methods must be used to assess drug supply chains, diagnostic systems, health worker training programs, community education activities, and facility-level patient flow management, among numerous other aspects of a health system and society.

Given these unique practice areas, we believe that IS will play an increasingly important role in global health agenda setting by rigorously identifying what evidence is needed to improve health care delivery, generating the evidence required, and seeing it through to actual evidence-based programming. We see important roles for IS practitioners within government health care systems, academic research institutions, non-governmental organizations, foundations, multilateral organizations, and government health offices. We hope that universities and research institutions continue to develop multidisciplinary IS training programs so that the number of IS practitioners continues to grow.

## Conclusion

IS has made great strides as a field of study, and there are a growing number of public health scientists with doctoral-level training in IS skills. At this junction of enormous health challenges and opportunities, we envision that IS will not only provide the necessary tools for global health professionals to address contemporary problems in context but also raise the bar for success in global health programs to come.

## Paper context

Implementation Science (IS) is a relatively new and innovative discipline that aims to address the gap between what is known and what is done in public health practice. As PhD students and graduates in one of the world's first doctoral programs in the science, we have a unique perspective on relevant training and global health practice. Necessary knowledge areas include population-level evidence generation, contextually appropriate intervention testing, and translation of evidence into policy and practice.
